# Effect of discontinuation of ticagrelor and switching-over to other P2Y12 agents in patients with acute coronary syndrome: a single-center real-world experience from India

**DOI:** 10.1186/s43044-020-00128-3

**Published:** 2021-01-11

**Authors:** Nagendra Boopathy Senguttuvan, Ramesh Sankaran, Yashasvi Rajeev, Devi Thaiyal, Angel Mathew, K. Dharsini, Divya Marcelene, Maria Jusler Kalsingh, Sujit Kumar Sahu, Aravind Sampath, K. J. Prem Kumar, Harikrishnan Parthasarathy, Amal Louis, Anand Gnanaraj, K. N. Reddy, K. A. Abraham

**Affiliations:** 1 Department of Cardiology, Sri Ramachandra Institute of Higher Education and Research, Chennai, Tamil Nadu India; 2 Adjunct Faculty-Indian Institute of Technology Madras, Chennai, Tamil Nadu India; 3 Department of Cardiology, Apollo Specialty Hospitals, Chennai, Tamil Nadu India; 4 Department of Cardiology, Jaswant Rai Specialty Hospitals, Chennai, Tamil Nadu India; 5 National Health Mission, Chennai, Tamil Nadu India

**Keywords:** Ticagrelor, Acute coronary syndrome, Bleeding, Clopidogrel, Switch over

## Abstract

**Background:**

Dual antiplatelet therapy is the current standard of care after acute coronary syndrome (ACS) and percutaneous coronary intervention (PCI). We intended to study the pattern of use of ticagrelor in patients with acute coronary syndrome undergoing PCI and the effect of switching over to other P2Y12 receptor inhibition on clinical outcomes.

**Results:**

All patients aged > 18 years who had been admitted with acute coronary syndrome and had been provided ticagrelor as the second antiplatelet agent were included as study participants. The primary outcome of the study was the composite outcome of death, recurrent myocardial infarctions, re-intervention, and major bleeding.

We studied 321 patients (54 female patients, 16.82%). The mean age of the patients was 56.65 ± 11.01 years. Ticagrelor was stopped in 76.7% on follow-up. It was stopped in 6.3%, 13.5%, 13.1%, 21.9%, and 45.1% of patients during the first month but after discharge, between first and third months, between 3 and 6 months, between 6 and 12 months, and after 12 months, respectively. In the majority of patients, ticagrelor was replaced by clopidogrel (97.9%). It was stopped according to the physician’s discretion in 79.3% of patients, whereas it was the cost of the drug that made the patient to get swapped to another agent in 18.6%. No difference in the primary composite outcome was observed between the groups where ticagrelor was continued post 12 months and ticagrelor was continued and ticagrelor was switched-over to another agent. Similarly, no difference in death, recurrent myocardial infarctions, re-interventions, or major bleeding manifestations was observed between the two groups.

**Conclusion:**

In patients with acute coronary syndrome who undergo PCI, we observed that early discontinuation of ticagrelor and switching over to other P2Y12 inhibitors after discharge did not affect clinical outcomes.

## Background

Coronary artery disease remains a major public health concern in India affecting people at their productive younger age. A recently published study from the state of Kerala estimated a prevalence of any CAD to be 12.5% (men 9.8%, women 14.3%) without any difference in urban and rural population [1]. Percutaneous coronary interventions (PCI) are increasingly used in our country. The role of PCI in patients with acute coronary syndrome especially ST-elevation MI is well established [2]. Anti-platelet drugs play a crucial role in the treatment of ACS. Dual antiplatelet therapy is the established mode of treatment in such scenarios. Until recently, it was clopidogrel that was available in treating such patients. At present, two more ADP receptor antagonists are available that include prasugrel and ticagrelor. It has been shown in TRITON TIMI-38, which was a randomized 13,608 patients with moderate-to-high-risk acute coronary syndromes with scheduled percutaneous coronary intervention to prasugrel or clopidogrel, that patients in the prasugrel arm had significantly reduced rates of ischemic events, including stent thrombosis. They also found that patients in the prasugrel arm had increased risk of major bleeding, including fatal bleeding [3]. Wallentin et al., in PLATO trial, studied 18,624 patients with acute coronary syndrome. They randomized the groups to clopidogrel or ticagrelor. They found that there was a significant reduction (absolute reduction of 1.9%) in the primary endpoint of the study that comprised of death from vascular causes, myocardial infarction, or stroke [4]. The rate of overall major bleeding was the same between the arms with an increase in the rate of non-procedure-related bleeding. Hence, it has been clearly shown in these two large randomized control trials that ticagrelor and prasugrel were superior to clopidogrel in patients with ACS [3, 4]. USFDA has approved both these agents in the treatment of patients with acute coronary syndrome who are getting intervened Though it is presumed that these drugs should act similarly in our Indian patients, there is a lack of indigenous evidence to prove the same. Similarly, the safety and efficacy of switching over from ticagrelor to clopidogrel is being addressed in many global studies. To our knowledge, no data is available from the sub-continent. Therefore, we intended to study the pattern of use of ticagrelor in patients with acute coronary syndrome undergoing PCI and the effect of switching over to other P2Y12 receptor inhibition on clinical outcomes in our study.

## Methods

Our study was a non-randomized, retrospective, single-center, observational study. It was an investigator-initiated, non-funded study. All patients aged > 18 years who had been admitted with acute coronary syndrome and had been provided ticagrelor as the second antiplatelet agent in the Department of Cardiology were included as study participants. The study was approved by Institutes Ethics committee, and patients provided their informed consent for the participation in the study. Study participants were identified from medical records through copyrighted software. Those patients who fulfilled the requirements were called individually by a research coordinator for detailed clinical assessment by their respective physicians. In case they were not able to make it in person, the necessary information was obtained from them over the phone. Baseline characteristics of the included but deidentified patients like age, sex, and presence of traditional risk factors for CAD including diabetes, hypertension, and dyslipidemia were studied. Patients were categorized into those with unstable angina, non-ST elevation myocardial infarction (NSTEMI), and ST-elevation myocardial infarction (STEMI). All available laboratory parameters were noted including electrocardiogram, transthoracic echocardiogram, angiographic findings, and interventional procedural along with clinically significant bleeding that required transfusions, reintervention, and recurrent myocardial infarction. Patients were specifically asked about their symptoms and adherence to antiplatelet agents. Details about the continuation of ticagrelor after procedure were also noted. Based on the same, they were divided into two groups. Group 1 where ticagrelor was discontinued and group 2 where it was continued. If it was stopped or swapped to a different p2Y12 inhibitor before one year, an attempt was made to analyze the reason for the same. The primary outcome of the study was the composite outcome of death, recurrent myocardial infarctions, re-intervention, and major bleeding requiring transfusions. We also intended to study the reason for the switch-over and its relation with clinical outcomes.

### Statistical analysis

Descriptive data were expressed in terms of ratio, proportion, or percentage; mean and median (interquartile range) were used for discrete quantitative data. Continuous variables were analyzed by *t* test. Categorical variables were analyzed by chi-squared test. A *p* value < 0.05 was considered significant. SPSS v20 (IBM) was used for statistical analysis.

## Results

We identified 336 patients. Out of this, we studied 321 patients (54 female patients, 16.8%) after the exclusion of 15 patients who could not be reached. The mean age of the patients was 56.65 ± 11.01 years (Table 1). The median duration of follow-up was 22 months (interquartile range 18). History of diabetes and hypertension were present in 56.7% and 52.3%, respectively. Prior history of CAD was present in 43.9%. Most of the patients had STEMI (47.4%) while 39.9% had unstable angina, and 12.8% had NSTEMI. The majority of the patients had good LV systolic function with an ejection fraction of > 55% in 57.9%. Mild LV dysfunction (LVEF45–55%), moderate LV dysfunction (30–45%), and severe LV dysfunction (< 30%) were present in 19.9%, 18.7%, and 3.4%, respectively. Nearly one third of the patients (35.2%) were taking aspirin before the index procedure. Some of the patients were receiving other antiplatelet agents that included 26.8% of patients with clopidogrel, 2.2% of patients with prasugrel, and 9.3% of patients with ticagrelor. More than 40% of individuals were using a statin. The history of prior bleeding was noted in 0.9%. The majority of the patients had left anterior descending artery (LAD) territory involvement. Most patients (98.8%) received stents, while 1.2% received plain old balloon angioplasty. Only two patients had received bare-metal stents. The mean stent used per patient was 1.3. Most of the patients had received Gp2b/3a inhibitor (82.9%). Non-culprit vessel intervention was done in 25.5% of patients during the index procedure or the same admission.

**Table 1:** Baseline characteristic of patient populations.

Characteristics	Number-321	*N* %
Age	56.65 ± 11.01 years	
Female	54	16.80
Diabetes mellitus	182	56.70
Insulin-dependent diabetes mellitus	27	8.40
Hypertension	168	52.30
Prior CAD	141	43.90
Unstable angina	128	39.90
NSTEMI	41	12.80
STEMI	152	47.40
EF ≤ 30%	11	3.40
EF ≥ 55%	186	57.90
EF-30–45%	60	18.70
EF-45–55%	64	19.90
Clopidogrel	86	26.80
Prasugrel	7	2.20
Aspirin	113	35.20
Ticagrelor	30	9.30
Statin	131	40.80
Prior bleeding	3	0.90
LAD	177	55.10
LCx-OM	51	15.90
RCA	88	27.40
Left main	5	1.60
Plain old balloon angioplasty (POBA)	4	1.20
DES	315	98.2
BMS	2	0.60
YES	266	82.90
Non-culprit vessel intervention	81	25.50
Recurrent MI	4	1.20
Re-intervention	3	0.90

**Fig. 1:**
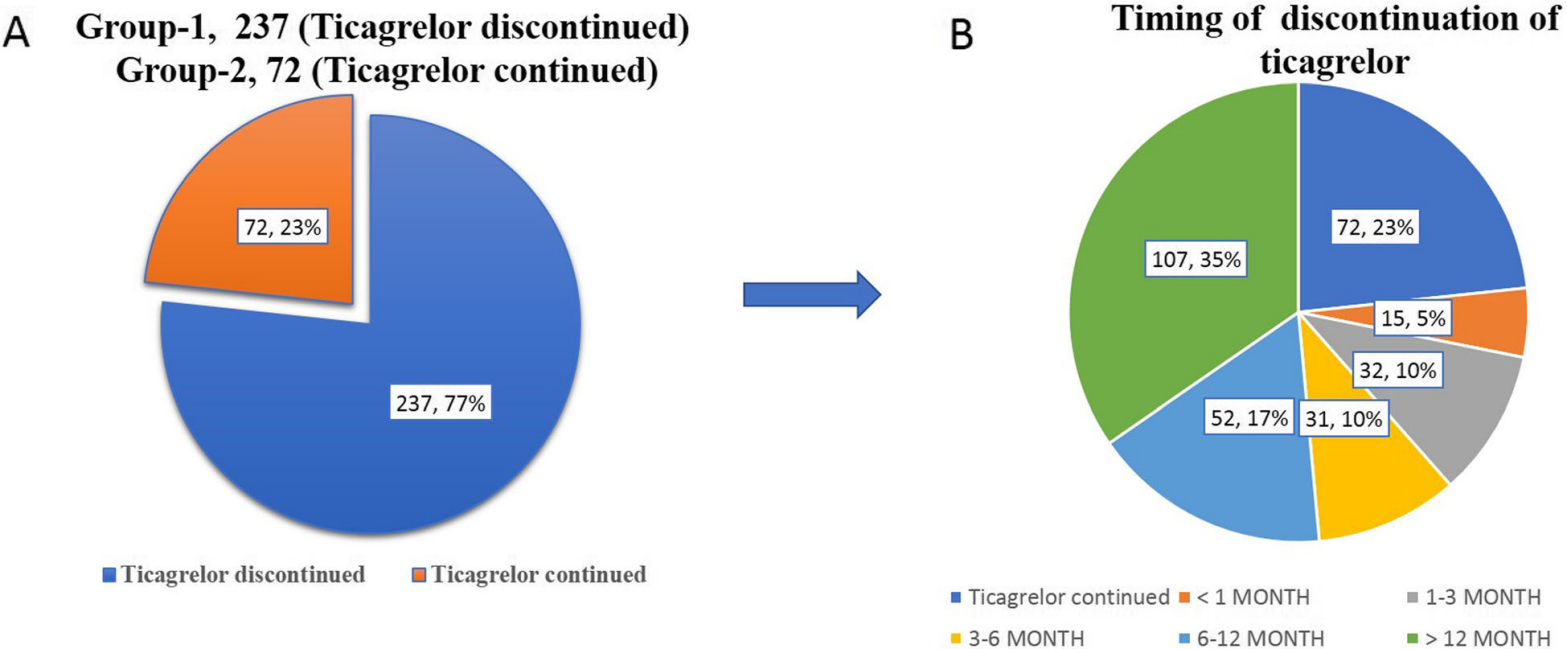
**a** Percentage of patients who discontinued ticagrelor. **b** Timing of discontinuation of Ticagrelor.

We excluded 12 patients who died before discharge for further analysis, as all patients were on ticagrelor during that period, i.e., before discharge from index hospital admission. This resulted in 309 patient populations for further analysis. No patient died during the follow-up study period**.** They were classified into group 1 (ticagrelor discontinued) and group 2 (ticagrelor continued) (Fig. 1 and Table 2). Ticagrelor was stopped and switched-over to other P2Y12 inhibitor in 76.7% of patients. Insulin-dependent diabetes status and hypertensive status were significantly more common in the group where ticagrelor was continued (Table 2). The primary composite event happened in 4.6% in the group where ticagrelor was discontinued and 8.3% where ticagrelor was continued (p-0.23, Table 3). Three out of 237 patients in whom ticagrelor was stopped early had recurrent MI while 1 out of 72 patients in the other arm had recurrent MI (P-non-significant). Similarly, no difference was seen in re-interventions and major bleeding between the studied groups (Table 3). The primary composite event happened in 5.5% in the group where ticagrelor was discontinued and 8.3% where ticagrelor was continued (p-0.229, Table 3). Three out of 237 patients in whom ticagrelor was stopped early had recurrent MI, while 1 out of 72 patients in the other arm had recurrent MI (P-non-significant). Similarly, no difference was seen in re-interventions and major bleeding between the studied groups (Table 3). There was no difference in the treated culprit vessel between the groups.

**Table 2:** Clinical outcomes in ticagrelor discontinued group and ticagrelor continued group.

	Group 1, n-237		Group 2, n-72		***p*** value
	*n*	*n*%	*n*	*n*%	
Female	44	18.60	7	9.70	0.077
Insulin-dependent diabetes mellitus	13	5.50	10	13.90	0.017
Prior bleeding	7	3.00	5	6.90	0.125
Hypertension	113	47.70	46	63.90	0.016
Prior CAD	101	42.60	31	43.10	0.947
Unstable angina	101	42.60	24	33.30	0.16
NSTEMI	25	10.50	12	16.70	
STEMI	111	46.80	36	50.00	
EF ≤ 30%	7	3.00	1	1.40	0.234
EF ≥ 55%	133	56.10	48	66.70	
EF-30–45%	44	18.60	14	19.40	
EF-45–55%	53	22.40	9	12.50	
Left anterior descending artery (LAD)	126	53.20	41	56.90	0.709
LCX-OM	41	17.30	10	13.90	
RCA	67	28.30	19	26.40	
Left main	3	1.30	2	2.80	
Recurrent MI	3	1.30	1	1.40	0.936
Re-intervention	3	1.30	0	0.00

**Table 3:** Comparison of baseline characteristics between ticagrelor discontinued and ticagrelor continued arms.

S. no.	Outcomes	Ticagrelor discontinued (237)	Ticagrelor continued (72)	*p* value
1	Composite outcome(n,%)	11 (4.6%)	6 (8.3)	0.23
2	Death	0	0	NA
3	Recurrent MI	3 (1.3%)	1 (1.4%)	0.94
4	Re-intervention	3 (1.2%)	0	0.34
5	Major bleeding	7 (3%)	5 (6.9)	0.12

**Fig. 2:**
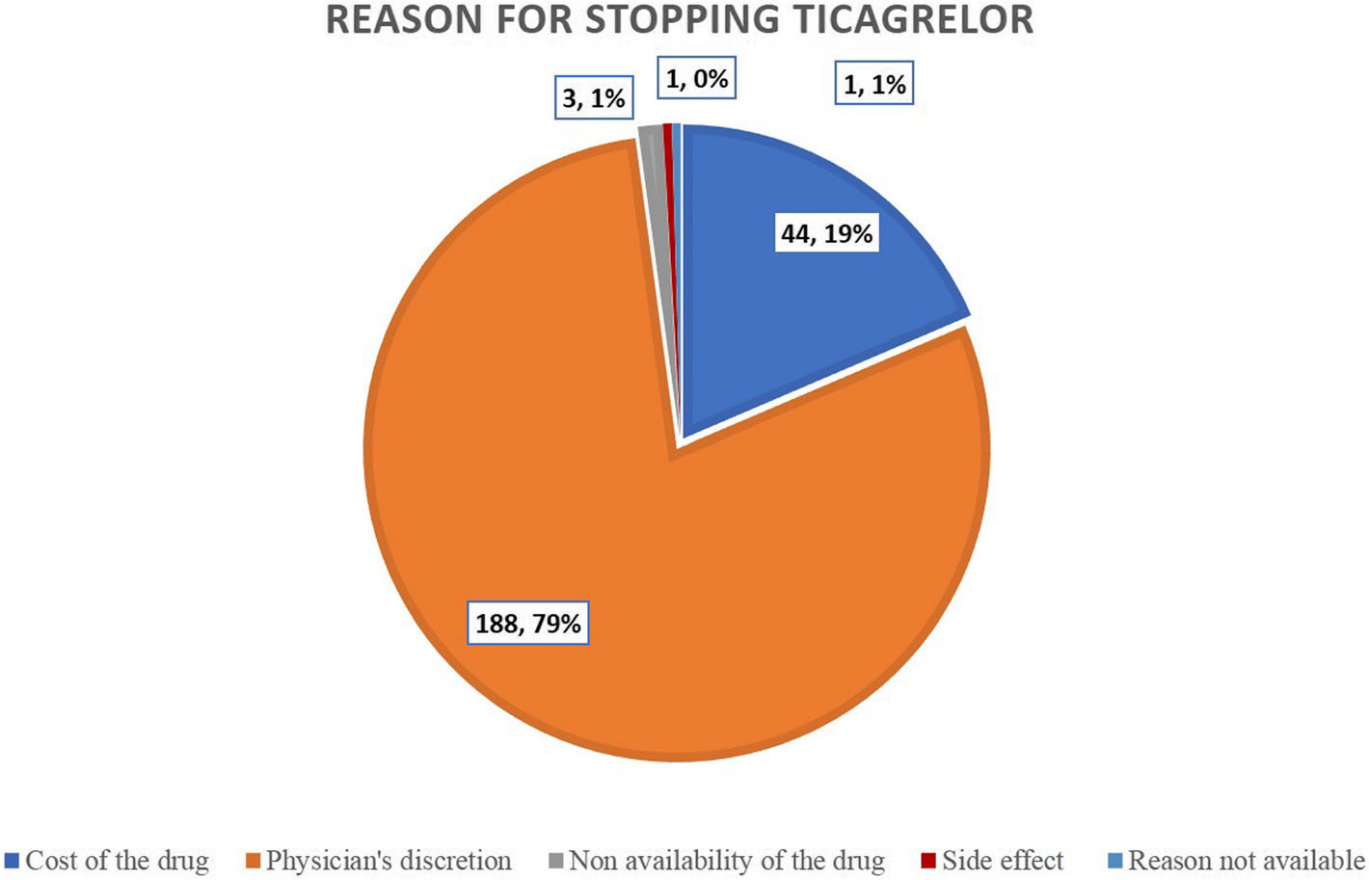
Reason for stopping ticagrelor and switching-over to other P2Y12 agents.

In the majority of those patients, it was stopped before 6 months. Period of stopping was classified empirically into five periods (Table 4 and Fig. 1). They were before the first month but after discharge from index hospitalization, between the first and third months, between 3 and 6 months, between 6 and 12 months, and after 12 months. It was stopped in 6.3%, 13.5%, 13.1%, 21.9%, and 45.1% of patients during the first month, between first and third months, between 3 and 6 months, between 6 and 12 months, and after 12 months, respectively (Table 4). We classified the basis for early dis-continuation into four possible causes (Table 3 and Fig. 2). They were (1) stopped due to the high cost of the drug, (2) stopped due to physician-based discretion, (3) stopped due to non-availability of drugs, and (4) stopped due to side effects. They were stopped according to physician’s discretion in 79.3% of patients, whereas it was the cost of the drug that made the patient to get swapped to another agent in 18.6% (Table 4). Only in a very small number of patients, it was stopped due to non-availability or side effect. Dyspnea was the reason to stop the drug in one patient during follow-up. In the majority of patients, ticagrelor was replaced by clopidogrel (97.9%), while in the remaining prasugrel was used. Most of the patients were loaded with a 300 mg loading dose of clopidogrel followed by 75 mg of maintenance dose while switching-over from ticagrelor. We assessed the effect various factors like diabetes, ACS, LV ejection fraction, reason for stopping ticagrelor, time of stopping ticagrelor, and nature of new drug added instead of ticagrelor on our primary outcome and found no significant association between any of the factors and the primary outcome (Table 5).

**Table 4:** Reason for discontinuation of ticagrelor and its timing

	Characteristics	Group-1, n-237	%
Reason for stopping ticagrelor	Reason not known	1	0.40
	Cost of the drug	44	18.60
	Physician’s discretion	188	79.30
	Non-availability of the drug	3	1.30
	Side effect	1	0.40
	Timing of discontinuation	Group 1, n-237	
Timing of discontinuation of ticagrelor	< 1 month	15	6.30
	1–3 months	32	13.50
	3–6 months	31	13.10
	6–12 months	52	21.90
	> 12 months	107	45.10

**Table 5:** Effect of various factors like diabetes, ACS, EF, reason for stopping ticagrelor, time of stopping ticagrelor, and nature of new drug added instead of ticagrelor on our primary outcome

	Primary outcome happened, N-17		Primary outcome did not happen, N-292		***p*** value
	*n*	*n*%	*n*	*n*%	
Diabetes	7	41.2	130	44.5	0.79
Acute coronary syndrome group					
USA	7	41.2	118	40.4	0.99
NSTEMI	2	11.8	35	12.0	
STEMI	8	47.1	139	47.6	
Ejection fraction					
EF ≤ 30%	0	0.0	8	2.7	0.8
EF ≥ 55%	11	64.75	170	58.2	
EF-30–45%	3	17.6	55	18.8	
EF-45–55%	3	17.6	59	20.2	
Culprit vessel					
Left anterior descending artery (LAD)	7	41.2	160	54.8	0.32
Circumflex (LCX)	2	11.85	49	16.8	
Right coronary artery (RCA)	8	47.1	78	26.7	
Left main or triple vessel disease	0	0.0	5	1.7	
Reason for stopping					0.70
Ticagrelor - not stooped	6	35.6	67	22.9	
Ticagrelor stopped - physician-based decision	1	5.9	1	14.7	
Ticagrelor stopped - cost	10	58.8	178	61.0	
Ticagrelor stopped - non-availability of drug	0	0.0	3	1.0	
Ticagrelor stopped - side effects	0	0.0	1	0.3	
Nature of drug that was used during switch-over from ticagrelor					
Ticagrelor continued	6	35.3	67	22.9	0.45
Clopidogrel	11	64.7	219	75.0	
Prasugrel	0	0.0	6	2.1	
Timing of ticagrelor stopped and event					
Not stopped	6	35.3	66	22.6	
Stopped < 1 month	2	11.8	13	4.5	0.44
Stopped 1–3 months	1	5.9	31	10.6	
Stopped 3–6 months	2	11.8	29	9.9	
Stopped 6–12 months	3	17.6	49	16.8	
Stopped > 12 months	3	17.6	104	35.6	

## Discussion

In this real-world single-center experience study, we observed early discontinuation of ticagrelor after discharge, and switching-over to other P2Y12 agents in patients with acute coronary syndrome did not affect clinical outcomes. It was found that ticagrelor was stopped early, i.e., before the end of the first year in the majority of patients. It happened more frequently after 6 months post PCI. Though the cost of ticagrelor remained an important factor in the discontinuation of the drug, it was stopped at the discretion of the physician in the majority of patients. Newer oral P2Y12 receptor blockers like ticagrelor and prasugrel have been shown to have increased bleeding risk as compared to clopidogrel [2, 3]. Similar to the PLATO trial, a large prospective registry from Sweden has shown better outcomes with ticagrelor as compared to clopidogrel [4]. Though few case reports from India attributed increased risk of bleeding to newer antiplatelet agents like ticagrelor [5], large observational studies have documented the safety of ticagrelor and prasugrel in the Indian subset of patients [6–8]. Similar to the other two studies from India, we observed ticagrelor to be safe in Indian patients. Major societal guidelines recommend continuing ticagrelor at least 12 months post-acute coronary syndrome interventions [9–11] based on the PLATO trial. In contrary to the above findings, the CHANGE-DAPT study has shown that ticagrelor was associated with increased events as compared to clopidogrel [12]. They categorized the period into the clopidogrel period (2012–2014) and ticagrelor period (2014–2015). They studied more than 2000 patients with ACS. The primary outcome of their study was net adverse cardiac and cerebral events (NACCE) that included all-cause death, any myocardial infarction, stroke, or major bleeding. They found that the 1-year NACCE rate was significantly higher during the ticagrelor period (5.1% vs. 7.8%; HR 1.53 [95% CI 1.08–2.17]; *p* = 0.02) that was attributed to more bleeding in these patients without any benefit in ischemic benefits. Cuisset et al. described the benefit of de-escalation of p2y12 inhibitors [13]. They studied 646 patients and found that switching DAPT strategy after a month of PCI in patients with acute coronary syndrome was superior to an unchanged DAPT strategy without any raise in ischemic events following ACS. In their subgroup analysis related to platelet reactivity study [14], they observed that benefit was seen in all groups irrespective of their platelet reactivity as assessed by vasodilator-stimulated phosphoprotein (VASP) assay. It was also found that greater benefits were seen in patients with lower platelet reactivity. In our study, we found no increased clinical events in patients who were continued on ticagrelor as compared to those who had been switched over to other P2Y12 agents. During swapping to clopidogrel, most of our patients were loaded with 300 mg of clopidogrel. In a recently presented study, it was found that loading with 600 mg of clopidogrel appeared a better strategy than 300 mg loading dosage [15].

In addition to the effect of early discontinuation of ticagrelor, we intended to study the reason behind the same. In the Paris registry, patients who had PCI were studied about the effect of cessation of DAPT, the reason behind the same, and its effect on clinical events [16]. We observed that ticagrelor was stopped in 237 patients (76.7%) of patients. They were stopped according to the physician’s discretion in 79.3% of patients, whereas it was the cost of the drug that made the patient to get swapped to other agents in 18.6%. Non-availability of the drug and the side effects were the reason for discontinuation in very few patients only. Clopidogrel was used as the replacement antiplatelet agent in the majority of patients. Ease of availability, long-term safety data, lesser bleeding complications, and cost of clopidogrel might have been the reason for swapping to clopidogrel instead of prasugrel. In one fifth of the patients, the cost of the drug was the reason behind the discontinuation. The availability of generic versions of ticagrelor may change this pattern of practice. We also observed no difference in outcomes of the patients according to the reason for stopping the drug.

## Conclusion

In patients with acute coronary syndrome who undergo PCI, we observed that early discontinuation of ticagrelor and switching over to other P2Y12 inhibitors after discharge did not affect the composite outcome of death, recurrent myocardial infarctions, re-intervention, and major bleeding requiring transfusions. More than 75%, ticagrelor was switched-over to another P2Y12 agent. Switching-over to other drug was performed frequently after 6 months post PCI. Around 80% of patients, the change was made by their physician. Clopidogrel was the primary replacement agent.

### Limitations

It was a retrospective study. Hence, all possible limitations due to retrospective study hold for this study. Though we arbitrarily categorized the reason for stopping the drug into different categories including the physician’s discretion, this might not be perfectly correct due to the retrospective nature of the study. Period of overlap between the groups cannot be excluded absolutely considering retrospective nature of the study. Events that happened in our study were few. That might be the reason for not having any significant difference between the groups. We could not contact 15 patients. It was a single-center experience. Whether it could be generalized to other centers remains a question.

## Data Availability

If required, we can submit the data sheet.

## Abbreviations

TRITON TIMI-38: Prasugrel versus clopidogrel in patients with acute coronary syndromes
PLATO: Ticagrelor versus clopidogrel in patients with acute coronary syndromes
CHANGE DAPT: Clopidogrel or ticagrelor in acute coronary syndrome patients treated with newer-generation drug-eluting stents
